# A NOVEL NATIONAL FAMILY CAREGIVER ADVOCACY TRAINING PROGRAM: LESSONS LEARNED

**DOI:** 10.1093/geroni/igad104.0827

**Published:** 2023-12-21

**Authors:** Heidi Donovan

**Affiliations:** University of Pittsburgh, Pittsburgh, Pennsylvania, United States

## Abstract

The 2022 National Strategy to Support Family Caregivers recommended 24 legislative or policy changes to improve the national caregiving landscape. With basic training, unpaid family caregivers (CG) can be powerful stakeholder advocates to drive policy change. In collaboration with national caregiver advocacy organizations, the University of Pittsburgh National Rehabilitation Research and Training Center on Family Support hosted a Family Caregiver Advocacy Program (FCAP) with the educational objectives of increasing CG motivation, self-efficacy for, and participation in policy advocacy. FCAP is a series of virtual workshops for family caregivers on caregiving-related policies, how laws are written and passed, how caregiver advocates can inform the policy-making process, how to contact and meet with legislators, and how to tell their story to advocate for change. Caregivers (n=68) enrolled in the inaugural FCAP workshops participated over a 3-month period to develop and implement group-selected advocacy products. FCAP participants ranged from 20-29 to 70+ years old, were primarily women (88%) caring for individuals who are aging (65%), have a physical disability (41%), have an intellectual / developmental disability (27%), and / or have a mental illness (27%). This presentation will report on the CG products and outcomes and plans for future iterations of FCAP based on lessons learned.

